# Genotypic and Phenotypic Characterization of *bla*_NDM–7_-Harboring IncX3 Plasmid in a ST11 *Klebsiella pneumoniae* Isolated From a Pediatric Patient in China

**DOI:** 10.3389/fmicb.2020.576823

**Published:** 2020-10-02

**Authors:** Chunhong Shao, Yingying Hao, Yong Wang, Meijie Jiang, Yan Jin

**Affiliations:** ^1^Clinical Laboratory of Shandong Provincial Hospital Affiliated to Shandong First Medical University, Jinan, China; ^2^Clinical Laboratory of Taian City Central Hospital, Tai’an, China

**Keywords:** NDM-7, *K. pneumoniae*, ST11, IncX3, China

## Abstract

NDM-7, a variant of New Delhi metallo-beta-lactamases (NDM), has the highest carbapenem-hydrolyzing activity. NDM-7-producing enterobacteria have been reported in many countries. In this study, we reported NDM-7 production in ST11 *Klebsiella pneumoniae* isolated from a boy hospitalized in the pediatric intensive care unit of a teaching hospital in China. The isolate exhibited resistance to β-lactam antimicrobials, quinolones, and trimethoprim/sulfamethoxazole, and it harbored *bla*_NDM–__7_, *bla*_CTX–M–__15_, *qnrA*, *qnrB*, and *qnrS*. The serotype of the isolated *K. pneumoniae* was assigned as K1, and it contained three virulence genes, including *kfuBC*, *uge*, and *fim*. The *bla*_NDM–__7_ gene was located on a conjugative IncX3 plasmid designated as pB14NDM-7. This plasmid was fully sequenced and compared with the available *bla*_NDM–__7_-harboring IncX3 plasmids. pB14NDM-7 contained a conserved genetic context of *ISkox3-umuD-IS26-ΔTn125*-IS5-*ΔTn125-IS3000-ΔTn2*. pB14NDM-7 showed 99% nucleotide identity and the same genetic context with three *bla*_NDM–__7_-harboring IncX3 plasmids obtained from *Escherichia coli* in China. Our results indicate that IncX3 plasmid may contribute to the prevalence of *bla*_NDM–__7_ in China. The high prevalence of NDM variants worldwide highlights the critical need for careful monitoring and control of the rapid dissemination of *bla*_NDM_.

## Introduction

The prevalence of carbapenem-resistant *Klebsiella pneumoniae* has increased and becomes a serious public health threat since the early 2000s. Several mechanisms are responsible for carbapenem resistance of *K. pneumoniae*, among which carbapenemase production remains the most clinically relevant (Pitout et al., 2015). The most common metallo-β-lactamases (MBLs) identified in *K. pneumoniae* are New Delhi metallo-beta-lactamases (NDM), while other metallolactamases are relatively rare in this species (Gregson et al., 2016). NDM was first described in Sweden in 2009 in a patient who had received medical care in India. Thereafter, it has been reported in more than 40 countries worldwide, even in cases with no epidemiological links with India, such as in the Balkan states or the Middle East (Yong et al., 2009; Pérez-Vázquez et al., 2019).

The gene encoding NDM is often carried by plasmids and is easily transferred to other microorganisms through horizontal gene transfer. Owing to this reason, the emergence of carbapenem-resistant strains of pathogenic microorganisms has increased rapidly (Rolain et al., 2010). NDM-1 is the most described NDM and has emerged as a global health threat. Similar to other MBLs, NDM-1 can hydrolyze all beta-lactams except aztreonam (Yong et al., 2009). Many NDM variants have evolved in enterobacteriaceae by single and/or double amino acid residue substitutions at different positions, and a total of 24 known variants of NDM have been identified so far (Khalid et al., 2020).

NDM-7 was first discovered in *Escherichia coli* in Germany in 2013 (Cuzon et al., 2013). NDM-7 contains Asp-130-Asn and Met-154-Leu substitutions and has the greatest carbapenem-hydrolyzing activity (Yoon et al., 2018). To date, NDM-7-producing enterobacteria have been reported in more than 10 countries (Solgi et al., 2017; Ahmad et al., 2018; Yoon et al., 2018; Mouftah et al., 2019). In China, NDM-7 has been detected and described in *E. coli* but not in *K. pneumoniae* (Wang et al., 2016; Bi et al., 2018; Hao et al., 2018; Xu and He, 2019). In this study, we isolated a ST11 *K. pneumoniae* harboring *bla*_NDM–__7_ from a boy hospitalized in the pediatric intensive care unit of a Chinese Hospital. Genotypic and phenotypic characterization of the IncX3 plasmid carrying *bla*_NDM–__7_ gene was performed. In 2015, an IncX3 plasmid carrying *bla*_NDM–__7_ was isolated from *E. coli* in the same hospital (Hao et al., 2018). To analyze the evolution of IncX3 plasmid carrying *bla*_NDM–__7_, we constructed a phylogenetic tree from 16 plasmids based on homologous proteins.

## Materials and Methods

### Bacterial Strains

Carbapenem-resistant *K. pneumoniae* was isolated from a 4-year-old boy who was diagnosed with autoimmune encephalitis and hospitalized in the pediatric intensive care unit of a teaching hospital in Shandong Province of China in 2018. We designated this isolate as B14. During hospitalization, arterial catheterization was performed. B14 was obtained from blood cultures 2 weeks after hospitalization; however, no pathogen was isolated from the sputum, cerebrospinal fluid, and ascites. The patient was treated with ceftriaxone before blood culture, following which his condition improved, and he was discharged before the antibiotic sensitivity results were obtained.

The patient had no history of traveling abroad. Informed consent was signed by the family member of the patient involved in this study. The methods in this study were approved by the Ethics Committee of Shandong Provincial Hospital and were carried out in accordance with the approved guidelines. The isolate was identified as *K. pneumoniae* using Vitek-2 compact system and confirmed using Vitek-MS system (BioMérieux, France). Phenotypic detection of carbapenemases was performed using carbapenem inactivation method (CIM) and EDTA-modified CIM (eCIM) test.

### Antibiotic Susceptibility Assay

Antimicrobial susceptibility testing was performed on Mueller Hinton agar plates using *E*-test strips (BioMérieux, France) (Table 1). The minimum inhibitory concentrations of tigecycline and colistin were determined using broth microdilution method (Bio-kont, China). *E. coli ATCC25922* and *K. pneumoniae ATCC700603* served as the quality controls. All antibiotics were administered according to the approved standard of the 2019 European Committee on Antimicrobial Susceptibility Testing breakpoint.^1^

**TABLE 1:** Antibiotic susceptibilities of B14 and its transconjugant (J14) (μg/ml).

	AMP	SAM	TZP	ATM	CRO	CAZ	FEP	FOX	IMP	MEM	ETP	CN	AK	CIP	LEV	SXT	TGC	COL
B14	≥256	≥256	≥256	≥256	≥256	≥256	≥256	≥256	≥32	≥32	≥32	≤0.016	≤0.016	≥32	≥32	≥32	0.5	0.5
J53 Azi^*R*^	≤0.016	≤0.016	≤0.016	≤0.016	≤0.016	≤0.016	≤0.016	≤0.016	≤0.02	≤0.02	≤0.02	≤0.016	≤0.016	≤0.02	≤0.02	≤0.02	0.125	0.125
J14	≥256	≥256	≥256	0.32	≥256	≥256	8	≥256	≥32	≥32	≥32	≤0.016	≤0.016	≤0.02	≤0.02	≤0.02	0.125	0.125

*AMP, ampicillin; SAM, ampicillin sulbactam; TZP, piperacillin/tazobactam; ATM, aztreonam; CRO, cefatriaxone; CAZ, ceftazidime; FEP, cefepime; FOX, cefoxitin; IPM, imipenem; MEM, meropenem; ETP, ertapenem; CN, gentamicin; AK, amikacin; CIP, ciprfloxacin; LEV, levofloxacin; STX, trimethoprim/sulfamethoxazole; TGC, tigecycline; COL, colistin.*

### PCR and DNA Sequence Analysis of Drug Resistance Genes, Serotype, and Virulence Genes

As described previously, a variety of antimicrobial resistance genes were screened using PCR and DNA sequencing (Zhu et al., 2016). These genes included carbapenem resistance genes (*bla*_KPC_, *bla*_SME_, *bla*_IMI_, *bla*_NMC_, *bla*_GES_, *bla*_IMP_, *bla*_VIM_, *bla*_GIM_, *bla*_SIM_, *bla*_SPM_, *bla*_NDM_, and *bla*_OXA–__48__like_), extended-spectrum β-lactamase genes (ESBLs) (*bla*_CTX–M_, *bla*_TEM_, and *bla*_SHV_), AmpC β-lactamase genes (*bla*_MOX_, *bla*_FOX_, *bla*_DHA_, *bla*_CIT_, and *bla*_EBC_), and plasmid-mediated quinolone resistance genes [*qnrA*, *qnrB*, *qnrC*, *qnrD*, *qnrS*, *qepA*, and *aac(6)-Ib-cr*]. Subsequently, the isolate was serotyped for K1, K2, K5, K20, K54, and K57 serotypes, and 12 virulence-associated genes (*rmpA*, aerobactin, *wcaG*, *ybtA*, *iucB*, *iroNB*, *ureA*, *uge*, *kfuBC*, *fim*, *wabG*, and *allS*) were screened using PCR as previously described (Wang et al., 2017).

### Multilocus Sequence Typing

Multilocus sequence typing (MLST) of *K. pneumoniae* was performed according to protocols available on the MLST Pasteur website^2^. Seven conserved housekeeping genes (*gapA*, *infB*, *mdh*, *pgi*, *phoE*, *rpoB*, and *tonB*) were amplified, sequenced, and compared with those in the MLST databases.

### Analysis of *bla*_NDM_-Carrying Plasmids

Conjugation was performed using the mixed broth method. Briefly, *E. coli J53* Azi^*R*^ was used as the recipient strain, and B14 served as the donor. Transconjugants were selected on Mueller Hinton agar supplemented with meropenem (0.5 μg/ml) and sodium azide (100 μg/ml). Antimicrobial susceptibility test of the transconjugant was carried out as described for the clinical strain. In order to evaluate the stability of the plasmid in the recipient *E. coli J53* after conjugation, antibiotic susceptibility test was performed after the recipient cells containing the plasmid were sequentially subcultured 20 times on blood agar plate without any antibiotics.

The size and amount of plasmids carried by the clinical isolate and transconjugant were evaluated using S1-pulsed-field gel electrophoresis (S1-PFGE) as previously described (Hao et al., 2018). The genome of *Salmonella* H9812 digested with *Xba*I was used as the marker.

### Plasmid Sequencing

The plasmid carrying *bla*_NDM–__7_ was defined as pB14NDM-7. To better understand the characteristics of pB14NDM-7, its complete sequence was determined. The plasmid was extracted and sequenced using an Illumina Hiseq platform and assembled using SOAPdenovo at MajorBio Co., (Shanghai, China). The gaps were closed through PCR and Sanger Sequencing at Sangon Biotech (Shanghai, China). The plasmid sequences were annotated using basic local alignment search tool (BLAST) against the non-redundant protein database. Plasmid Finder was used for detection and typing of the plasmid.

### Phylogenetic Tree Construction

The plasmids carrying *bla*_NDM–__7_ were retrieved from National Center for Biotechnology Information (NCBI) database for the query “NDM-7 and plasmid.” Out of 49 plasmids, 15 belonged to the IncX3 incompatibility group. The sequence of pB14NDM-7 was compared with the 15 *bla*_NDM–__7_-harboring IncX3 plasmids. The plasmid sequences were annotated for encoded proteins using Prodigal, a prokaryote genome annotation tool^3^. The STAG algorithm of OrthoFinder was used to construct a phylogenetic tree from 16 plasmids based on homologous proteins.

## Results

### Resistance Profile of B14 Strain

Clinical *K. pneumoniae* B14 was identified as an MBL-producing strain using carbapenem inactivation method (CIM) and EDTA-modified CIM (eCIM) test. B14 was resistant to aztreonam, ampicillin, ampicillin/sulbactam, piperacillin/tazobactam, cephalosporins, carbapenems, quinolones, and trimethoprim/sulfamethoxazole and susceptible to aminoglycosides, colistin, and tigecycline (Table 1).

### MLST, Serotype, Resistance, and Virulence Genotyping

PCR amplification and sequencing confirmed that B14 harbored *bla*_NDM–__7_ and *bla*_CTX–M–__15_. These two genes were responsible for the resistance to β-lactam antibiotics. In addition, B14 carried *qnrA*, *qnrB*, and *qnrS*, which may lead to quinolones resistance. However, high-level fluoroquinolone resistance was probably mediated by chromosomal mutations in *gyrA* and/or *parC* genes. MLST revealed that the sequence type of B14 was ST11. Based on PCR amplification results, the serotype of B14 was determined to be K1. Furthermore, B14 contained three virulence genes, which included *kfuBC*, *uge*, and *fim*.

### Transferability and Stability of Plasmid

*bla*_NDM–__7_-harboring plasmid of B14 was successfully transferred into *E. coli J53* by conjugation. The transconjugant was identified as *E. coli* using Vitek-2 compact system and confirmed using Vitek-MS system. The presence of *bla*_NDM–__7_ in the transconjugant was confirmed using PCR. The transconjugant was resistant to carbapenems and cephalosporin but susceptible to aztreonam, aminoglycosides, quinolones, colistin, and tigecycline. After 20 passages, the antibiotic susceptibility patterns of the transconjugant showed no changes. S1-pulsed-field gel electrophoresis showed that B14 harbored two plasmids, and the transconjugant J14 contained a single plasmid, which was approximately 46 kb (Supplementary Figure S1).

### Characterization of *bla*_NDM–__7_-Harboring Plasmid

**FIGURE 1:**
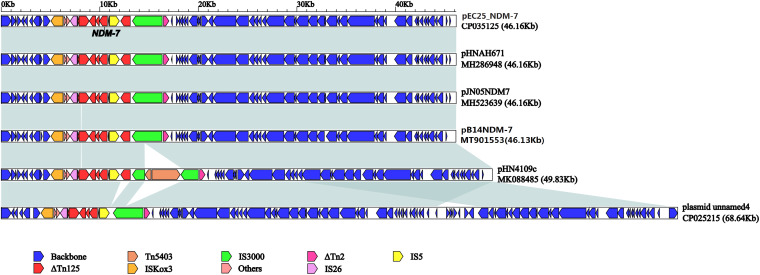
Linear comparison of pB14NDM-7 with five *bla*_NDM–7_-harboring IncX3 plasmids from China. A linear comparison was carried out for the complete DNA sequences of pB14NDM-7 (MT901553, this study), pJN05NDM7 (MH523639), pHNAH671 (MH286948), pEC25_NDM-7 (CP035125), pHN4109c (MK088485), and plasmid unnamed 4 (CP025215). Genes, mobile elements, and other features are colored based on function classification. Shading denotes regions of homology (>95% nucleotide identity).

pB14NDM-7 is a 46,133-bp plasmid belonging to the IncX3 incompatibility group. The complete sequence of pB14NDM-7 was submitted to GenBank under accession number MT901553. In pB14NDM-7, *bla*_NDM–__7_ was preceded by *ISkox3-umuD-IS26-ΔTn125 (ΔdsbC-trpF-bleMBL)* in the upstream region and succeeded by IS5-*ΔTn125-IS3000-ΔTn2* in the downstream region (Figure 1).

**FIGURE 2:**
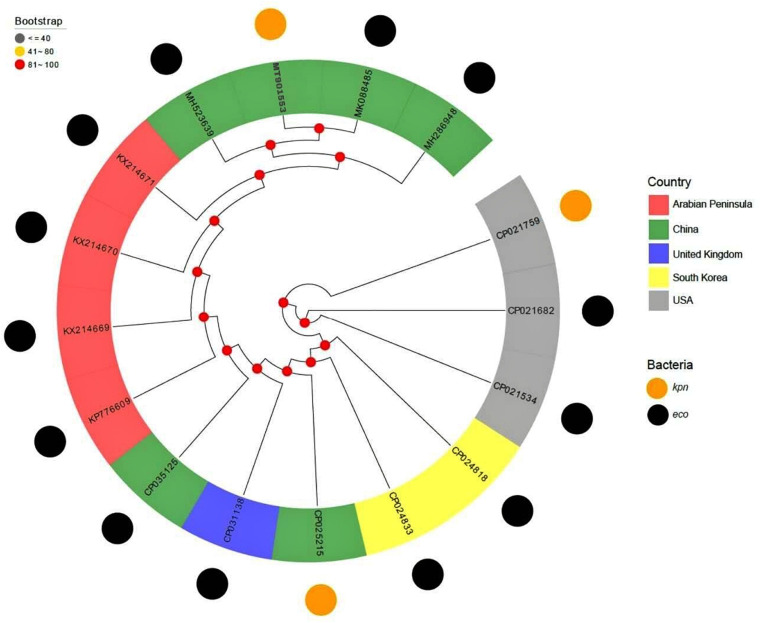
A phylogenetic tree of 16 *bla*_NDM–7_-harboring IncX3 plasmids.

The full published sequences of 15 IncX3 plasmids harboring *bla*_NDM–__7_ were downloaded and compared with pB14NDM-7 (Figure 2). Only 3 of the 16 plasmids were isolated from *K. pneumoniae*, and the other 13 were from *E. coli.* Sequence alignments revealed more than 80% homology among the 16 plasmids. The sequence of pB14NDM-7 showed more than 99% homology with three plasmids, including pHN4109c (accession no. MK088485), pJN05NDM-7 (accession no. MH523639), and pHNAH671 (accession no. MH286948). All four plasmids were from China, with pB14NDM-7 being the only plasmid to be isolated from *K. pneumoniae.* To determine the detailed structural differences among six *bla*_NDM–__7_-harboring IncX3 plasmids from China, including pB14NDM-7 (MT901553, this study), pJN05NDM7 (MH523639), pHNAH671 (MH286948), pEC25_NDM-7 (CP035125), pHN4109c (MK088485), and plasmid unnamed 4 (CP025215), additional linear comparative genomics analysis of these six plasmids was performed by BLAST. The six plasmids showed similar genomic contents. pB14NDM-7, pJN05NDM7, pEC25_NDM-7, and pHNAH671 had the same genetic structure. In plasmid pHN4109c, the insertion of Tn5403 resulted in the interruption of IS3000 (Figure 1).

Plasmids pB14NDM-7 and pJN05NDM-7 were from the same hospital and differed only in gene *dsbC*. pJN05NDM-7 is a 46,161-bp circular IncX3-type plasmid. In *dsbC* gene, there was a 60-bp repetitive sequence in the region between the positions 8084 and 8144, corresponding to the 8023–8083 regions in plasmid pJN05NDM-7. In addition, there was a 88-bp deletion in pB14NDM-7 corresponding to the 8085–8172 region in gene *dsbC* of plasmid pJN05NDM-7.

## Discussion

NDM-7-producing *K. pneumoniae* are relatively rare as compared to NDM-7-producing *E. coli.* NDM-7 was first reported in *K. pneumoniae* in 2014 in Minnesota (Lee et al., 2014). Herein, we described the emergence of ST11 *K. pneumoniae* carrying *bla*_NDM–__7_ on IncX3 plasmid in China. Although NDM-7 production has been reported in diverse clones of *K. pneumoniae*, including ST138, ST273, ST278, ST437, and ST654 (Shankar et al., 2019), our study is the first to report the presence of *bla*_NDM–__7_ in ST11 *K. pneumoniae* in China. This strain was isolated from a 4-year-old boy with no history of traveling abroad.

The *K* serotypes and phenotypes are responsible for the invasive nature of certain *K. pneumoniae.* Serotypes K1, K2, K4, and K5 are highly virulent and may cause severe infections in humans (Wang et al., 2017). The serotype of B14 was found to be K1. The carbapenem-resistant K1 *K. pneumoniae* can cause infections at multiple sites, including liver abscesses, pneumonia, meningitis, and blood stream infections. Additionally, B14 contained three virulence genes, which included *kfuBC*, *uge*, and *fim*. ST11 is the major sequence type of hypervirulent carbapenem-resistant *K. pneumoniae* from Asia, especially China (Zhao et al., 2019). Therefore, it is speculated that B14 has certain virulence traits. However, it is gratifying that the patient’s condition improved, following which he was discharged. As this study was retrospective, we could not perform in-depth analysis of the patient’s surrounding environment and the source of the strain.

B14 was highly resistant to broad spectrum cephalosporins, monobactam, and carbapenems. However, it is known that NDM does not decompose aztreonam (Paul et al., 2017). Therefore, resistance of B14 to aztreonam in the present study might be due to the coexistence of *bla*_CTX–M–__15_ gene that encodes extended spectrum β-lactamase. In addition, PCR results showed that B14 also carried quinolone-resistance genes, including *qnrA*, *qnrB*, and *qnrS*. Conjugation test revealed that only *bla*_NDM–__7_ could be horizontally transferred, and the sequencing results showed that plasmid IncX3 harbored by B14 does not carry antimicrobial resistance genes other than *bla*_NDM–__7_. This finding corroborates the results of Hao et al. (2018).

IncX3 plasmids carrying *bla*_NDM_ variants have been increasingly reported all over the world in recent years. A previous study has proved that IncX3 plasmid can transfer *bla*_NDM_ between different enterobacterial species over a wide range of temperatures (Liu et al., 2019). IncX3 has also been reported to be an important carrier of *bla*_NDM–__7_ (Paul et al., 2017). In order to further understand the evolutionary relationship of *bla*_NDM–__7_-harboring IncX3 plasmid, we downloaded the nucleotide sequences of 15 IncX3 plasmids from NCBI and analyzed their homology with pB14NDM-7. The results showed that all the 16 plasmids were closely related to each other, suggesting parallel evolution of these plasmids. It should be noted that pB14NDM-7 showed 99% nucleotide identity with three plasmids obtained from *E. coli* in China, which suggests that IncX3-type plasmids are popular vectors in mediating dissemination of *bla*_NDM–__7_ in China (Wang et al., 2016; Bi et al., 2018).

In plasmids, genes are frequently associated with mobile genetic elements such as transposons (Tn) and insertion sequences (IS). pB14NDM-7 contained a conserved genetic context of *ISkox3-umuD-IS26-ΔTn125*-IS5-*ΔTn125-IS3000-ΔTn2.* The *bla*_NDM_ genetic structure is common in enterobacteriaceae for the horizontal transfer of *bla*_NDM_ and has been reported in the transmission of *bla*_NDM–__7_ and *bla*_NDM–__5_ (Zhang et al., 2016; Guo et al., 2019). In addition, plasmids pB14NDM-7 and pJN05NDM-7 were from the same hospital. However, pJN05NDM-7 was isolated from *E. coli*, and the two patients were not in the same hospital area. These two plasmids differed only in gene *dsbC*. Disulfide bond formation is a crucial step in the folding process of a protein and is catalyzed by bacterial proteins of the Dsb system. DsbC is responsible for rearranging incorrect disulfides introduced between cysteine residues (Banaś et al., 2020).

## Conclusion

In summary, our study described the emergence of *bla*_NDM–__7_ in ST11 *K. pneumoniae* in China for the first time. Our results indicate that IncX3 plasmid may contribute to the prevalence of *bla*_NDM–__7_ in China. The high prevalence of NDM variants worldwide highlights the critical need for careful monitoring and control of the rapid dissemination of *bla*_NDM_.

## Data Availability Statement

The datasets presented in this study can be found in online repositories. The names of the repository/repositories and accession number(s) can be found in the article/Supplementary Material.

## Ethics Statement

The studies involving human participants were reviewed and approved by the Ethics Committee of Shandong Provincial Hospital. Written informed consent to participate in this study was provided by the participants’ legal guardian/next of kin. Written informed consent was obtained from the minor(s)’ legal guardian/next of kin for the publication of any potentially identifiable images or data included in this article.

## Author Contributions

YJ designed the experiments and revised the manuscript. CS carried out the experiments and wrote the manuscript. YH analyzed the data. YW and MJ contributed to experiment conception. All authors contributed to the article and approved the submitted version.

## Conflict of Interest

The authors declare that the research was conducted in the absence of any commercial or financial relationships that could be construed as a potential conflict of interest.

## Footnotes

1 www.eucast.org/clinical-breakpoint

2 http://www.pasteur.fr/recherche/genopole/PF8/mlst/K. pneumoniae.html

3 http://compbio.ornl.gov/prodigal/

## Abbreviations

MBL: metallo- β-lactamases
MLST: multilocus sequence typing
NDM: New Delhi metallo-beta-lactamases.
