# Supplementary figures and images for: Genotypic and Phenotypic Characterization of blaNDM–7-Harboring IncX3 Plasmid in a ST11 Klebsiella pneumoniae Isolated From a Pediatric Patient in China
Source: Front Microbiol. 2020 Oct 2;11:576823. doi: 10.3389/fmicb.2020.576823 (PMC7566911; doi:10.3389/fmicb.2020.576823)

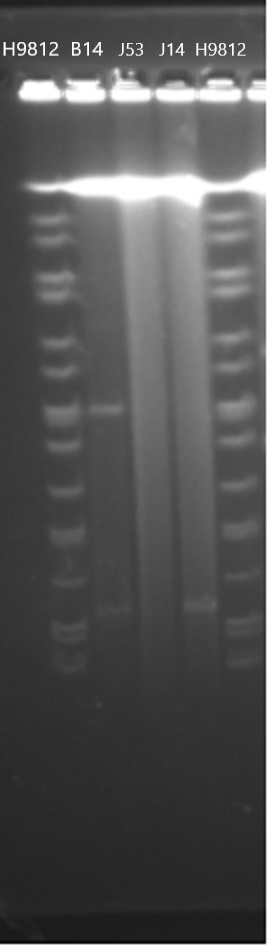

Supplement: Supplementary file 2 [file Image_1.jpg]
